# DNA barcoding of formalin-fixed aquatic oligochaetes for biomonitoring

**DOI:** 10.1186/s13104-016-2140-1

**Published:** 2016-07-13

**Authors:** Régis Vivien, Benoit J. D. Ferrari, Jan Pawlowski

**Affiliations:** Swiss Centre for Applied Ecotoxicology (Ecotox Centre), Eawag/EPFL, Lausanne, Switzerland; Department of Genetics and Evolution, University of Geneva, Geneva, Switzerland

**Keywords:** Aquatic oligochaetes, Formalin fixation, *COI* amplification and sequencing, Biomonitoring

## Abstract

**Background:**

Oligochaetes are valuable bioindicators of the quality of watercourse and lake sediments. The morphological identification of aquatic oligochaetes is difficult, prompting the development of new molecular oligochaete indices based on DNA barcoding and Next-generation sequencing of sorted specimens. In general, the samples for DNA barcoding are fixed in absolute ethanol. However, in the case of aquatic oligochaetes, this medium is not appropriate as it can induce a modification of specimen abundances and of the composition of communities. Therefore, we investigated the possibility to amplify and sequence aquatic oligochaetes fixed in formalin for a short time. We performed guanidine extraction and polymerase chain reaction (PCR) amplification/sequencing of the cytochrome c oxydase I (*COI*) gene on tissue fragments fixed in formalin for different periods of time (from 1 h to 1 week) and in ethanol.

**Results:**

The large majority of aquatic oligochaete specimens fixed in formalin for up to 1 week could be successfully amplified and all obtained sequences were of high quality. The amplification and sequencing success rate of formalin-fixed samples and ethanol-fixed samples was similar. These results suggest that formalin fixation of aquatic oligochaete tissues for a short time does not cause serious damages to DNA and inhibit PCR amplification.

**Conclusion:**

The possibility to fix aquatic oligochaetes with formalin before genetic analyses is very promising for diversity monitoring, for construction of a comprehensive DNA barcode library and for development of an index based on Next-generation sequencing analysis of samples composed of sorted specimens.

**Electronic supplementary material:**

The online version of this article (doi:10.1186/s13104-016-2140-1) contains supplementary material, which is available to authorized users.

## Background

Oligochaetes represent an important group of benthic invertebrates and constitute good bioindicators of the quality of sediments in watercourses and lakes [1, 2]. The morphological identification of aquatic oligochaetes is difficult and not possible for most specimens present in a sample, as many species can be identified only in a mature form. This problem can be solved by the use of DNA barcodes to identify oligochaetes. Next-generation sequencing technology, which allows a rapid and simultaneous processing of large sample sets, is a promising tool to assess the biological quality of aquatic ecosystems [3]. A reference library of *COI* barcodes was developed for specimens collected in the Geneva area [4] and the capacity of Next-generation sequencing to recover the composition of aquatic oligochaete communities was tested on samples composed of sorted specimens [5].

In general, absolute ethanol is considered the best medium to preserve DNA of tissue samples and is strongly recommended for the specimens that will be processed for DNA barcoding [6]. However, ethanol is not appropriate for aquatic oligochaetes as it often results in fragmentation and disintegration of specimens and so affects abundance and diversity estimates [7]. Moreover, ethanol fixation modifies the shape of specimens (contraction) so that, at the sorting step (stereo microscope), the distinction of anterior and posterior parts is sometimes difficult and the identification of specimens at the family level is often difficult.

In contrast, formalin is a good fixative and preserves optimally the composition of oligochaete communities and the shape of specimens. This medium is not considered as appropriate to conserve DNA but the duration of exposure of specimens to formalin has a great influence on the amount and quality of DNA obtained [6, 8, 9].

Other factors related to chemical composition of formalin and to the conditions of fixation can influence DNA yield after formalin fixation. For example, low-pH formalin damages more DNA than neutral buffered formalin and fixation and storage of specimens in formalin at 4 °C causes less degradation than storage at room temperature [8]. In addition, ethanol is a good medium to remove formalin from specimens and so it is important to transfer specimens to ethanol after formalin fixation [8]. Finally, Timm and Martin [10] recommend preservation of aquatic oligochaetes in strong ethanol (80–96 %) at −20 °C to avoid DNA degradation.

Here, we tested the possibility to extract and amplify DNA on samples composed of aquatic oligochaete tissue fragments fixed in formalin for a short time. For each of 69 oligochaete specimens, we prepared tissue fragment samples fixed in formalin for different durations (from 1 h to 7 days) before their transfer to absolute ethanol and a tissue fragment fixed only in absolute ethanol. We compared extraction and amplification success of formalin-fixed tissue fragments and of ethanol-fixed tissue fragments. We also sequenced several samples fixed in formalin to verify that high-quality DNA and full-length sequences were obtained.

## Methods

### Preparation of samples

Sediment samples were collected in 2015 in the Geneva area in the Hermance River (46.29618°N 6.24996°E) and in the canton of Vaud in the Sorge River (46.52266°N 6.57357°E). Sieving was performed the same day as the collection or up to 3 days after collection. After sieving, the samples were stored at 4 °C until the sorting of oligochaete specimens. The sorting was performed either the same day or a few days later (max 10 days after sieving). Two, three or four parts of similar sizes of each live specimen were cut. One part was put directly in absolute ethanol, while the other parts were stored in 6 % of low-pH (pH = 2.8–4) formalin for several durations (1, 2 h, 1, 2, 3, 4, 6 or 7 days). The specimens kept in formalin for more than 2 h were stored at 4 °C. At the end of each storage duration, the parts in formalin were transferred into tap water for few seconds and then into absolute ethanol. Once in ethanol, each part was immediately kept at −20 °C until extraction process (for 2 days–2 months). The anterior part of several specimens was fixed and preserved in formalin or absolute ethanol for identification by compound microscope.

### DNA extraction, PCR and sequencing

The total genomic DNA was extracted using the guanidine thiocyanate method described by Tkach and Pawlowski [11]. A fragment of 658 base pairs of the *COI* gene was amplified using LCO 1490 and HCO 2198 primers [12]. Each PCR was performed in a total volume of 20 μl containing 0.6 Unit of Taq polymerase (Roche), 2 μl of the 10× buffer (Roche) containing 20 mM of MgCl_2_, 0.5 μl of each primer (10 mM each), 0.4 μl of a mix containing 10 mM of each dNTP (Roche) and 0.8 μl of template DNA of undetermined concentration. The PCR process comprised an initial denaturation step at 95 °C for 5 min, followed by 35 cycles of denaturation at 95 °C for 40 s, annealing at 44 °C for 45 s and elongation at 72 °C for 1 min, with a final elongation step at 72 °C for 8 min. The PCR products were then directly and bi-directionally Sanger sequenced on an ABI 3031 automated sequencer (Applied Biosystems) using the same primers and following the manufacturer’s protocol. The raw sequence editing and the generation of contiguous sequences were accomplished using CodonCode Aligner (CodonCode Corporation). Multiple sequence alignments were automatically generated using Muscle v3.8.31 [13] as implemented in Seaview v.4.4.0 [14].

### Oligochaete identification

Specimens were identified at the family, sub-family or species level, either by stereo microscope/compound microscope analysis or by genetic analysis. For the identification by compound microscope, the anterior parts were cleared in an acid lactic/glycerol solution and mounted between slide and coverslip in a permanent coating solution composed of lactic acid, glycerol and polyvinylic alcohol (Mowiol 4–88). The genetic analysis was performed by constructing a phylogenetic tree with sequences of this study and sequences of our *COI* database [4] using the neighbour-joining method as implemented in Seaview v.4.4.0 [14], with 1000 bootstrap replicates. A 10 % threshold of *COI* divergence was applied to segregate between species [4].

## Findings

Sixty-nine specimens were sorted and the numbers of tissue samples fixed in formalin for ≤2 h, 1–3 days and 4–7 days (and then in ethanol) and fixed in ethanol only were 60, 44, 38 and 69, respectively. Out of these 69 specimens, we identified 52 individuals (Additional file 1). 26 specimens belonged to Lumbriculidae (9 Lumbriculidae sp., 17 *Stylodrilus heringianus* Claparède, 1862), 15 to Naidinae (9 *Nais elinguis* Müller 1774, 6 Naidinae sp.), 9 to Tubificinae (3 *Psammoryctides barbatus* (Grube, 1861), 1 *Tubifex tubifex* Müller 1774, 2 *Limnodrilus hoffmeisteri* Claparède, 1862, 1 *Limnodrilus udekemianus* Claparède, 1862, 1 *Limnodrilus claparedeanus* Ratzel, 1868, 1 Tubificinae sp.) and 2 to Haplotaxidae (*Haplotaxis gordioides* (Hartmann 1821).

We observed that almost all specimens fixed in formalin for different periods of time and in ethanol could be PCR amplified (Table 1). The intensities of PCR bands of almost all formalin-fixed samples were sufficient for Sanger sequencing and the percentage of bands of weak intensity was low. The amplification success rate of samples fixed in formalin for ≤2 h and in ethanol was identical, while it was slightly lower for samples fixed in formalin for 1–3 and 4–7 days than for samples fixed in ethanol.

**Table 1:** Number of successfully amplified specimens/total number of analysed specimens, for formalin and ethanol fixation

Formalin ≤2 h	Formalin 1–3 days	Formalin 4–7 days	Ethanol
59/60	42/44	36/38	68/69

Fourteen samples fixed in formalin for 2 h to 3 days and 16 samples fixed in formalin for 6–7 days were sequenced (28 specimens in total). These samples corresponded to different PCR band intensities. Sequencing was also performed on ethanol-fixed samples from the same specimens. With the exception of one sample fixed in formalin, all samples could be sequenced and all the sequences obtained were whole and of high quality (Table 2).

**Table 2:** Number of successfully sequenced specimens/total number of sequenced specimens, for formalin and ethanol fixation

Formalin 2 h to 3 days	Formalin 6–7 days	Ethanol
14/14	15/16	28/28

## Discussion

The amplification and sequencing of aquatic oligochaete tissues fixed in formalin for up to 1 week were successful. We observed no clear difference in amplification success rate between formalin-fixed samples and ethanol-fixed samples. The results also suggest that the amplification and sequencing success of aquatic oligochaetes fixed in formalin is not species-dependant.

Most articles or reports on recovering of DNA from formalin-fixed samples concern specimens of museums fixed in formalin over long periods of time or for which the duration fixation in formalin is unknown. The yield of sequencing of long-term formalin-fixed specimens is generally low, as formalin strongly affects the structure of DNA, provoking among others DNA fragmentation and nucleotide alteration [9, 15, 16]. The sequences obtained in our study were of high quality. So we can conclude that 1 week in formalin is not sufficient to cause DNA damages or to inhibit Taq polymerase binding. Baird et al. [6] showed that formalin preservation of four invertebrate species, including one oligochaete species, for up to 20 days followed by transfer of specimens in ethanol 70 %, yielded high-quality sequences.

A good amplification yield was obtained despite the fact that we used unbuffered formalin. Our results show that unbuffered formalin can be used successfully for amplification of oligochaete tissues fixed in formalin for up to 1 week. Bucklin and Allen [9] also observed that short-time storage (until 40 days) of a copepod in unbuffered formalin, followed by a transfer of specimens to absolute ethanol, did not affect its amplification. But these authors also observed that long storage of zooplancton in unbuffered formalin could not be amplified. These results suggest that the use of buffered formalin is especially important when specimens are stored in formalin for a long time.

The recovery of DNA after short time fixation of oligochaete tissues in formalin showed in our study is promising for diversity monitoring, for construction of a comprehensive DNA barcode library and for development of an index based on Next-generation sequencing analysis of sorted specimens, as the use of formalin instead of ethanol makes possible to sort and sequence all specimens present in a sample.

The perspectives of this work are to compare DNA yield after fixation of aquatic oligochaete tissues with buffered and unbuffered formalin and to test Next-generation sequencing performance to recover the composition of species on samples composed of formalin-fixed specimens.

## Additional file

10.1186/s13104-016-2140-1 Performed analyses (formalin fixation for 1 h to 7 days and ethanol fixation) and taxonomic identification per sample. X = analysis performed. Following each taxon name is indicated in brackets how the specimen was identified: 1 = with stereo microscope, 2 = with compound microscope, 3 = with genetic analysis.

## Abbreviations

*COI*: cytochrome c oxydase subunit I
PCR: polymerase chain reaction
