# Supplementary material for: DNA barcoding of formalin-fixed aquatic oligochaetes for biomonitoring
Source: BMC Res Notes. 2016 Jul 13;9:342. doi: 10.1186/s13104-016-2140-1 (PMC4944268; doi:10.1186/s13104-016-2140-1)
Supplement: Supplementary file 1 — 10.1186/s13104-016-2140-1 Performed analyses (formalin fixation for 1 h to 7 days and ethanol fixation) and taxonomic identification per sample. X = analysis performed. Following each taxon name is indicated in brackets how the specimen was identified: 1 = with stereo microscope, 2 = with compound microscope, 3 = with genetic analysis. [file 13104_2016_2140_MOESM1_ESM.doc]

Additional file 1. Table S1: Performed analyses (formalin fixation for 1 h to 7 days and ethanol fixation) and taxonomic identification per sample. X = analysis performed. Following each taxon name is indicated in brackets how the specimen was identified : 1 = with stereo microscope, 2 = with compound microscope, 3 = with genetic analysis

| **No sample** | **1 h** | **2 h** | **1 d** | **2 d** | **3 d** | **4 d** | **6 d** | **7 d** | **ethanol** | **identification** |
| --- | --- | --- | --- | --- | --- | --- | --- | --- | --- | --- |
| 872 | X | X |  |  | X |  |  |  | X | Lumbriculidae sp (2) |
| 873 | X | X |  |  | X |  |  |  | X | Lumbriculidae sp (2) |
| 874 | X | X |  |  | X |  |  |  | X | *Stylodrilus heringianus* Claparède, 1862 (3) |
| 875 | X | X |  |  | X |  |  |  | X | Lumbriculidae sp (1) |
| 876 | X | X |  |  | X |  |  |  | X | Lumbriculidae sp (2) |
| 877 | X | X |  |  | X |  |  |  | X | *Stylodrilus heringianus* Claparède, 1862 (2) |
| 878 | X | X |  |  | X |  |  |  | X | Lumbriculidae sp (2) |
| 879 | X | X |  |  | X |  |  |  | X | *Stylodrilus heringianus* Claparède, 1862 (2, 3) |
| 880 | X | X |  |  | X |  |  |  | X | *Stylodrilus heringianus* Claparède, 1862 (3) |
| 881 | X | X |  |  | X |  |  |  | X | Lumbriculidae sp (2) |
| 882 | X | X |  |  |  |  | X |  | X | *Stylodrilus heringianus* Claparède, 1862 (2, 3) |
| 883 | X | X |  |  |  |  |  |  | X | Lumbriculidae sp (2) |
| 884 | X | X |  |  |  |  | X |  | X | Lumbriculidae sp (2) |
| 885 | X | X |  |  |  | X |  |  | X | *Haplotaxis gordioides* (Hartmann 1821) (2, 3) |
| 886 | X | X |  |  |  | X |  |  | X | *Stylodrilus heringianus* Claparède, 1862 (2) |
| 887 | X | X |  |  |  | X |  |  | X | *Stylodrilus heringianus* Claparède, 1862 (2) |
| 888 |  | X |  |  | X |  |  |  | X | *Nais elinguis* Müller, 1774 (2, 3) |
| 889 | X | X |  |  | X |  |  |  | X | *Stylodrilus heringianus* Claparède, 1862 (2) |
| 890 | X | X |  |  | X |  |  |  | X | *Stylodrilus heringianus* Claparède, 1862 (2) |
| 891 | X | X |  |  | X |  |  |  | X | Lumbriculidae sp (2) |
| 892 | X | X |  |  |  |  |  | X | X | *Haplotaxis gordioides* (Hartmann 1821) (2, 3) |
| 893 |  |  |  |  |  |  |  | X | X | *Nais elinguis* Müller, 1774 (2) |
| 894 |  |  |  |  |  |  |  | X | X | *Nais elinguis* Müller, 1774 (2) |
| 895 |  | X | X |  |  |  |  | X | X | *Nais elinguis* Müller, 1774 (3) |
| 896 |  | X | X |  |  |  |  | X | X | *Stylodrilus heringianus* Claparède, 1862 (2, 3) |
| 897 |  | X | X |  |  |  |  | X | X | *Stylodrilus heringianus* Claparède, 1862 (2, 3) |
| 898 |  | X | X |  |  |  |  | X | X | Naidinae sp (1) |
| 899 |  | X | X |  |  |  |  | X | X | *Stylodrilus heringianus* Claparède, 1862 (2, 3) |
| 900 |  |  | X |  |  |  |  | X | X | *Nais elinguis* Müller, 1774 (3) |
| 901 |  | X |  |  |  |  | X |  | X | Naidinae sp (1) |
| 902 |  | X |  |  |  |  | X |  | X | Naidinae sp (1) |
| 903 |  | X |  |  |  |  | X |  | X | *Nais elinguis* Müller, 1774 (3) |
| 904 |  | X |  |  |  |  | X |  | X | *Nais elinguis* Müller, 1774 (3) |
| 905 |  | X |  |  |  |  | X |  | X |  |
| 906 |  | X |  |  |  |  | X |  | X |  |
| 907 |  | X |  |  |  |  | X |  | X | *Psammoryctides barbatus* (Grube, 1861) (2, 3) |
| 908 |  |  |  |  | X |  |  | X | X | Naidinae sp (1) |
| 909 |  |  |  |  | X |  |  | X | X | Naidinae sp (1) |
| 910 |  |  |  |  | X |  |  | X | X | *Nais elinguis* Müller, 1774 (3) |
| 911 |  |  |  |  | X |  |  | X | X | *Nais elinguis* Müller, 1774 (3) |
| 912 |  |  |  |  | X |  |  | X | X | Naidinae sp (1) |
| 913 |  |  |  |  | X |  |  | X | X | *Psammoryctides barbatus* (Grube, 1861) (2, 3) |
| 914 |  | X |  |  | X |  |  | X | X | *Stylodrilus heringianus* Claparède, 1862 (2) |
| 915 |  | X |  | X |  |  |  | X | X |  |
| 916 |  | X |  | X |  |  |  | X | X | *Stylodrilus heringianus* Claparède, 1862 (2, 3) |
| 917 |  | X |  | X |  |  |  | X | X | *Stylodrilus heringianus* Claparède, 1862 (2, 3) |
| 918 |  | X |  | X |  |  |  | X | X | *Stylodrilus heringianus* Claparède, 1862 (2, 3) |
| 919 |  | X |  | X |  |  |  | X | X | *Psammoryctides barbatus* (Grube, 1861) (2, 3) |
| 920 | X | X |  |  |  |  |  |  | X | *Limnodrilus claparedeanus* Ratzel, 1868 (2) |
| 921 | X | X | X |  |  |  |  |  | X |  |
| 922 | X | X | X |  |  |  |  |  | X |  |
| 923 |  | X |  |  | X |  |  |  | X |  |
| 924 | X |  |  |  | X |  |  |  | X |  |
| 925 | X | X |  |  |  |  |  |  | X |  |
| 926 | X | X |  |  |  |  |  |  | X |  |
| 927 | X | X |  |  |  |  |  |  | X |  |
| 928 | X | X | X |  |  |  |  |  | X |  |
| 929 | X | X | X |  |  |  |  |  | X | *Limnodrilus udekemianus* Claparède, 1862 (2, 3) |
| 930 | X | X | X |  |  |  |  |  | X |  |
| 931 | X | X |  | X |  |  |  |  | X |  |
| 932 | X | X |  | X |  |  |  |  | X |  |
| 933 | X | X |  | X |  |  |  |  | X | *Limnodrilus hoffmeisteri* Claparède, 1862 (3) |
| 934 | X | X |  | X |  |  |  |  | X |  |
| 935 | X | X |  | X |  |  |  |  | X | *Limnodrilus hoffmeisteri* Claparède, 1862 (3) |
| 936 | X | X |  |  |  | X |  |  | X | *Stylodrilus heringianus* Claparède, 1862 (3) |
| 937 | X | X |  |  |  | X |  |  | X |  |
| 938 | X | X |  |  |  | X |  |  | X | *Tubifex tubifex* (Müller, 1774) (3) |
| 939 | X | X |  |  |  | X |  |  | X | Tubificinae sp (3) |
| 940 | X | X |  |  |  | X |  |  | X |  |
